# Radon Diffusion Coefficients of Barrier Materials: A Critical Review of Measurement, Modeling, and Laboratory-to-Field Interpretation

**DOI:** 10.3390/ma19214648

**Published:** 2026-10-31

**Authors:** Chutima Kranrod, Yasutaka Omori, Rawiwan Kritsananuwat, Phachirarat Sola, Eka Djatnika Nugraha, Shinji Tokonami

**Affiliations:** 1 Institute of Radiation Emergency Medicine, Hirosaki University, Hirosaki 036-8564, Aomori, Japan; kranrodc@hirosaki-u.ac.jp (C.K.); ys-omori@hirosaki-u.ac.jp (Y.O.); 2 Department of Nuclear Engineering, Faculty of Engineering, Chulalongkorn University, Patumwan, Bangkok 10330, Thailand; rawiwan.kr@chula.ac.th; 3 Thailand Institute of Nuclear Technology (Public Organization), Ongkharak, Nakhon Nayok 26120, Thailand; phachirarats@tint.or.th; 4 Research Center of Nuclear Safety, Metrology and Quality Technology, Research Organization of Nuclear Technology (ORTN), National Research and Innovation Agency (BRIN), South Tangerang 15314, Indonesia; ekad001@brin.go.id

**Keywords:** Radon-222, diffusion coefficient, effective diffusivity, apparent diffusivity, radon barrier, boundary condition, measurement uncertainty, Peclet number, thoron-220

## Abstract

Radon-resistant membranes and other barrier materials are widely used to limit soil-gas entry into buildings, yet published radon diffusion coefficients are difficult to compare because the reported measurand, boundary conditions, leakage treatment, and test conditions differ among studies. This critical review examines how these coefficients are measured, modeled, and compared, and why laboratory values do not necessarily predict installed barrier performance. The material diffusion coefficient, the effective coefficient of porous media, and the apparent coefficient obtained by fitting experimental data are distinguished, and the coefficient affected by each influencing factor is identified. Steady-state and transient methods are compared in terms of the quantity obtained, test duration, and dominant sources of bias. Illustrative calculations show that a fixture leakage conductance of only 10^−11^ m^3^ s^−1^ can inflate the apparent coefficient of a highly resistant membrane several-fold, that the Peclet number of an intact specimen can be far below unity while a sub-millimeter crack is advection-dominated, and that defects occupying less than 10^−5^ of the barrier area can dominate radon entry. Gas-side resistance, model-form uncertainty, mixed-mode transport, and thoron-specific constraints are also discussed. Harmonized reporting, joint and penetration testing, interlaboratory comparison, durability assessment, and laboratory-to-field validation are identified as priorities.

## Simple Summary

Radon entering buildings from the ground increases the risk of lung cancer, and barrier materials are used to limit its entry. Published radon diffusion coefficients for these materials are difficult to compare because studies define and measure them in different ways. This review explains which quantity each method actually measures, shows with simple calculations how small leaks, air-pressure differences, and installation defects can distort test results or dominate radon entry, and explains why a low coefficient measured in the laboratory does not guarantee good performance in a building. It closes with practical recommendations for researchers, testing laboratories, manufacturers, designers, and standards bodies.

## 1. Introduction

Radon (^222^Rn) is a naturally occurring radioactive noble gas formed by the decay of radium-226 in the uranium-238 series [1]. Its gaseous nature permits migration from rocks and soils into buildings through diffusion and pressure-driven flow [2]. Inhaled short-lived radon progeny are the principal contributors to the associated lung dose. Radon is a major contributor to natural radiation exposure [3]. The World Health Organization (WHO) identifies radon as the second leading cause of lung cancer after smoking; the estimated proportion of lung cancers attributable to radon varies with national radon concentrations and smoking prevalence [4].

The WHO recommends an annual-average indoor radon reference level of 100 Bq m^−3^. Where this cannot be achieved under prevailing country-specific conditions, the reference level should not exceed 300 Bq m^−3^ [4]. The ICRP also recommends an upper reference level of 300 Bq m^−3^ for dwellings [5]. These reference levels support national radon-control programs and the evaluation of mitigation measures, including barrier systems.

The primary sources of indoor radon are: (1) soil and rock beneath building foundations, which typically contribute the majority of indoor radon, and (2) building materials themselves, particularly those containing elevated levels of natural radionuclides [2,3]. Radon enters buildings through several pathways, including cracks in concrete floors and walls, gaps around service pipes, construction joints, and through the porous structure of building materials [6]. The rate of radon entry is influenced by multiple factors, including soil radon concentration, soil permeability, pressure differentials between soil and indoor air, and the integrity of the building envelope.

One way to limit soil-gas entry is to install a continuous barrier beneath or around the building envelope [7,8]. Material selection is often based on a reported radon diffusion coefficient, but this number alone does not establish installed performance. Membrane thickness, radon solubility or partitioning, joints, penetrations, punctures, and pressure-driven bypass flow must also be considered. A low coefficient measured on a pristine coupon may therefore coexist with poor performance of the installed system.

The term diffusion coefficient is used inconsistently in the literature [9,10,11]. For a homogeneous phase it may denote a molecular or material property; for porous media it usually denotes an effective coefficient that already incorporates pore geometry and phase occupancy; and in inverse experiments it may be an apparent coefficient that absorbs unmodeled leakage, boundary resistance, detector response, or advection. These quantities are not interchangeable. This review uses explicit subscripts and states the associated model and boundary conditions wherever possible.

In this review, barrier material is the general term, membrane denotes a thin continuous sheet, and barrier system includes seams, penetrations, substrates, and seals. The term transient is used in preference to non-steady state except when reproducing a source title.

This critical review addresses three questions: (1) how radon diffusion coefficients of barrier materials are measured, and which quantity each method actually delivers; (2) how transport models, boundary conditions, leakage, and uncertainty treatment affect the inferred coefficient and its comparability among studies; and (3) why a coefficient measured on an intact laboratory specimen does not necessarily predict the performance of an installed barrier system. Rather than ranking materials by pooled coefficients, the review evaluates the evidence by measurement model and transport mechanism, appraises the sources of scatter in reported values, and uses order-of-magnitude calculations to make the practical consequences of leakage, advection, gas-side resistance, and installation defects explicit. Detector, numerical, and metrological aspects are treated only insofar as they influence the inferred coefficient.

### Scope and Literature Selection

This narrative review was based on a structured search of Scopus, Web of Science Core Collection, ScienceDirect, and Google Scholar, supplemented by backward and forward citation tracking and by ISO and national standards catalogues. Search strings combined “radon”, “^222^Rn”, “thoron”, or “^220^Rn” with “diffusion coefficient”, “diffusion length”, “permeability”, “membrane”, “barrier”, “waterproofing”, “coating”, “building material”, or “radon entry”. Foundational works were included irrespective of date; for measurement methods and barrier data, publications up to July 2026 were considered. Studies were retained if they reported an experimental or modeled radon or thoron diffusion coefficient, permeability, or diffusion length of a barrier or building material; described an apparatus, boundary-condition treatment, or analysis method used to infer such a coefficient; or provided assembly-, room-, or building-scale evidence relevant to interpreting laboratory coefficients. Conference abstracts without methodological detail, studies of other gases without a stated link to radon, and duplicate reports were excluded. Because measurands and test conditions were too heterogeneous for meta-analysis, results were not pooled (Section 6). Related applied fields, namely the metrology of radon in water [12] and numerical modeling of radon entry into houses [13], were consulted where they inform partitioning, calibration, or building-scale interpretation.

## 2. Fundamental Principles of Radon Diffusion

### 2.1. Fick’s Laws of Diffusion [14]


(1) $$J=-D\frac{\partial C}{\partial x}$$

Here *J* is the activity flux. The concentration and coefficient must refer to the same phase and volume convention. For a homogeneous material, *C* = *C*_m_ and *D* = *D*_m_. Porous-medium conservation additionally requires the storage convention specified in Section 2.4.

For a single homogeneous phase with no advection or internal source, activity conservation and Fick’s law give:

(2) $$\frac{\partial C}{\partial t}=\frac{\partial }{\partial x}\left(D\frac{\partial C}{\partial x}\right)-\mathrm{\lambda C}$$

where the term −*λC* accounts for radioactive decay of radon (half-life = 3.82 days).

### 2.2. Steady-State Versus Transient Conditions

Steady-state conditions occur when the radon concentration distribution within the material and the exhalation rate become time-independent. Under one-dimensional steady-state conditions, Equation (2) simplifies to:

(3) $$\frac{d}{\mathrm{dx}}\left(D\frac{\mathrm{dC}}{\mathrm{dx}}\right)-\mathrm{\lambda C}=0$$

For constant *D*, this further simplifies to:

(4) $$D\frac{d^{2}C}{dx^{2}}-\mathrm{\lambda C}=0$$

For a homogeneous slab with fixed surface concentrations, the slowest relaxation time is [λ + π^2^*D/d*^2^]^−1^. Chamber mixing, source evolution, and detector response can introduce additional time scales. Equilibrium should therefore be defined using a stated concentration or flux tolerance. Membrane-specific studies illustrate the dependence of measurement duration on the apparatus and specimen [15,16,17].

Transient methods use time-dependent concentration data without requiring equilibrium. The duration needed to identify transport parameters depends on specimen thickness, diffusion and partition coefficients, leakage, chamber dynamics, and detector response. Fitting can use analytical or numerical solutions, provided that the initial conditions and measurement model are represented adequately [15,16,17,18].

### 2.3. Definitions of Material, Effective, and Apparent Diffusion Coefficients

Three quantities should be separated.

Molecular or material diffusion coefficient (*D_m_*). This coefficient describes molecular transport within a single homogeneous material phase and appears in Fick’s law as

*J_m_* = −*D_m_*∇*C_m_* (5)

where *C_m_* is the radon concentration within the material. In a dense polymer, *D_m_* characterizes the mobility of dissolved radon atoms in the polymer phase. Gas-to-polymer equilibrium is described separately by the dimensionless partition coefficient,

(6) $$K=\frac{C_{m}}{C_{g}}$$

where *C_g_* is the adjacent gas-phase concentration. When these definitions are used, the permeability coefficient is then *P* = *KD_m_*. Thus, a flux written using gas-phase concentrations depends on both *K* and *D_m_*; reporting *P* as though it were *D_m_* is incorrect [19,20]. Temperature and polymer composition can affect both *D_m_* and *K* [19].

Effective diffusion coefficient (*D_eff_*). In this review, the porous-medium coefficient is defined per unit bulk cross-sectional area and is also denoted D_bulk_. It incorporates connected gas-filled porosity and pore geometry. One commonly used expression is [21,22,23]

(7) $$D_{\mathrm{eff}}=D_{0}\frac{\varepsilon_{g}}{\tau }$$

Here *ε*_g_ is the connected gas-filled porosity, *τ* is a dimensionless tortuosity factor under the stated convention, and *D*_0_ is the free-air radon diffusion coefficient. Thus *D*_eff_ = *D*_bulk_ in Equation (7). Other definitions use *τ*^2^ or additional connectivity factors. The corresponding pore-volume (interstitial) coefficient is *D*_pore_ = *D*_bulk_/*ε*_g_ for constant *ε*_g_, so that *D*_bulk_ = *ε*_g_*D*_pore_. These coefficients must not be interchanged. Aqueous storage and gas–water partitioning are discussed in Section 5.2.2.

Apparent diffusion coefficient (*D*_app_). This is the value inferred by fitting an experimental concentration–time curve or measured steady-state flux using a specified transport and chamber model [15,16,17,24]. It can be interpreted as a material property only when the model adequately represents the specimen, boundary conditions, measurement system, and experimental apparatus. Unmodeled seal leakage, edge bypass, stagnant air boundary layers, sorption, chamber nonuniformity, background concentration, detector response, or pressure-driven airflow may be incorporated unintentionally into *D*_app_ [11,16,17,25]. Every experimentally reported value should therefore identify the fitted model, imposed boundary conditions, leakage and background corrections, detector-response treatment, environmental conditions, and parameter-estimation procedure.

These distinctions are especially important for joints and penetrations. A coefficient obtained from a membrane specimen containing seams, joints, penetrations, or damaged regions is an assembly-level apparent parameter rather than the intrinsic material diffusion coefficient of the intact membrane matrix [26,27].

### 2.4. Diffusion-Decay-Advection Equation and Regime Criterion

For gas-phase activity concentration *C_g_*, gas-filled porosity *ε_g_*, Darcy velocity *u_D_*, and source S_bulk_ per unit bulk volume, conservation in an isotropic porous medium is [21,23]

(8) $$\frac{\partial \left(\varepsilon_{g}C_{g}\right)}{\partial t}+\nabla \cdot \left(u_{D}C_{g}\right)=\nabla \cdot \left(D_{\mathrm{bulk}}\nabla C_{g}\right)-\lambda \varepsilon_{g}C_{g}+S_{\mathrm{bulk}}\;$$

Here D_bulk_ includes gas-filled porosity and has units of m^2^ s^−1^. For constant *ε_g_*, dividing Equation (8) by *ε_g_* gives the pore-volume form with *v_g_* = *u_D_*/*ε_g_* and D_pore_ = D_bulk_/*ε_g_*. In the absence of advection and sources, this divided equation has the form of Equation (2). The corresponding Peclet number is

(9) $$\mathrm{Pe}=\frac{\left|u_{D}\right|L_{c}}{D_{\mathrm{bulk}}}=\frac{\left|v_{g}\right|L_{c}}{D_{\mathrm{pore}}}$$

where *L_c_* is a stated characteristic length, commonly the specimen thickness. A diffusion-only model is generally defensible when *Pe* ≪ 1 [28] and no preferential path is present. Mixed transport should be modeled when *Pe* is of order unity, whereas *Pe* ≫ 1 indicates advection-dominated transport. Because velocity in a narrow crack can be much greater than velocity in the intact matrix, a specimen-average *Pe* can conceal locally advective joints [26,27,29,30]. The pressure difference across the specimen should therefore be measured or bounded, and the velocity definition (Darcy velocity or pore-gas velocity) must be stated [21,29,31].

Order-of-magnitude example. For Darcy flow driven by a pressure difference Δ*p* across a homogeneous specimen of thickness *d*, *u*_D_ = *k*_g_Δ*p*/(*μ*_g_*d*), where *k*_g_ is the gas permeability and *μ*_g_ the gas viscosity. With *L*_c_ = *d*, Equation (9) then becomes

(10) $$\mathrm{Pe}=\frac{k_{g}\Delta p}{\mu_{g}D_{\mathrm{bulk}}}$$

so that the specimen-average Peclet number is independent of thickness and is set by the ratio of permeability to diffusivity. For an intact concrete specimen with *k*_g_ = 10^−16^ m^2^, *D*_bulk_ = 10^−8^ m^2^ s^−1^, *μ*_g_ = 1.8 × 10^−5^ Pa s, and Δ*p* = 10 Pa (a typical indoor–soil pressure difference [29,30]), *Pe* ≈ 6 × 10^−3^, and a diffusion-only model is defensible. The same specimen subjected to a pump-induced imbalance of 50 Pa, or a more permeable material with *k*_g_ = 10^−14^ m^2^ at 10 Pa, gives *Pe* ≈ 0.03 and 0.6, respectively; in the latter case, a diffusion-only fit would assign advective transfer to *D*_app_. By contrast, a 0.5 mm wide planar crack through a 0.1 m slab carries a mean gas velocity of about 6 × 10^−2^ m s^−1^ at Δ*p* = 5 Pa (plane Poiseuille flow, *v* = *w*^2^Δ*p*/(12*μ*_g_*L*)), giving a local *Pe* ≈ 5 × 10^2^ with *D*_0_ as the reference diffusivity. Specimen-average and local Peclet numbers can therefore differ by about five orders of magnitude.

For experiments, the criterion converts a measured or bounded pressure difference into a quantitative test of the diffusion-only assumption; its limitation is that it presupposes homogeneous permeability and cannot detect localized pathways unless they are resolved. For numerical simulation, the analogous mesh Peclet number, *Pe*_h_ = |*u*|*h*/(2*D*) for element size *h*, indicates whether standard Galerkin or central-difference schemes will oscillate (*Pe*_h_ > 1), and hence whether local mesh refinement or a stabilized advection scheme is required [32,33].

### 2.5. Selecting Governing Equations for Dense, Porous, and Mixed-Mode Materials

The governing equation should follow from the dominant transport mechanism rather than from the material label. For dense, non-porous polymers and bitumens, solution–diffusion transport is described by *D*_m_ and *K* (or *P* = *KD*_m_) with partition conditions at both interfaces (Equations (5), (6) and (13)). For porous materials with connected gas-filled porosity, gas-phase transport is described by Equation (8) with *D*_bulk_ and *ε*_g_, together with a Darcy term when the pressure difference is not negligible. Composite and mixed-mode materials, including polymer-modified cementitious coatings, filled polymers, coated porous substrates, and laminated systems, combine both mechanisms, and the arrangement of the phases determines the model. When the phases are layered in series (for example, a continuous polymer film over a mortar substrate), the area-normalized resistances add, *R*_total_ = Σ*d*_i_/*P*_i_, provided that the partition convention is consistent at every interface and decay within each layer is negligible [14,34]; otherwise, the layered diffusion–decay problem must be solved with interfacial partition conditions. When a continuous dense matrix is interrupted by connected pores, microcracks, or pinholes (parallel pathways), the fluxes add, *J* ≈ (1 − *φ*)*J*_dense_ + *φJ*_pore_, where *φ* is the area fraction of the connected porous pathway; because *J*_pore_ can exceed *J*_dense_ by several orders of magnitude, even a small *φ* controls the result [35]. When isolated inclusions are dispersed in a continuous matrix, effective-medium approximations are appropriate, and multiphase formulations for porous media containing gas, water, and sorbed phases provide the general framework [36].

Simple diagnostics help to identify the dominant mechanism: the thickness dependence of the flux (proportional to 1/*d* for a homogeneous layer, weaker for defect-controlled transport); its sensitivity to an imposed pressure difference (negligible for solution–diffusion, appreciable for connected porosity); its temperature dependence (strongly activated for polymers, Equation (26)); and the shape of the transient breakthrough curve, which may show two time constants when fast and slow pathways coexist. When these diagnostics are ambiguous, single- and dual-pathway models should be fitted to the same data and compared by residual analysis and an information criterion (Section 4.4). The wide range and comparatively high values reported for polymer–cement coatings reported for polymer–cement coatings (Section 6) are consistent with mixed-mode behavior and should be interpreted as assembly-level *D*_app_.

## 3. Experimental Methods for Measuring Radon Diffusion Coefficients

### 3.1. General Experimental Setup

The empirical determination of radon diffusion coefficients typically relies on a dual-chamber configuration designed to isolate and track gas migration. This setup generally integrates the following core components:

- Source Chamber: A reservoir connected to a radon source, such as a radium solution, radon generator, or radon-emitting material. Its concentration history must be measured or modeled; a constant concentration should not be assumed solely from the presence of a source.
- Receiver Chamber: A volume in which transported radon is measured. Record its initial concentration and background, together with the initial radon loading and conditioning history of the specimen.
- Sample Interface: A specialized holder that secures the barrier material between the two chambers. Precise, airtight sealing at this interface is critical to prevent leakage and ensure that all detected radon has passed through the sample matrix.
- Detection Systems: Continuous monitors (for example, ionization chambers or electrostatic-collection alpha spectrometers), scintillation cells, or integrating solid-state nuclear track detectors, selected according to the required time resolution and concentration range (Section 3.2.5).
- Environmental Monitoring: Given that radon mobility is sensitive to external factors, integrated sensors are used to regulate or record temperature and humidity throughout the duration of the test.

The measured quantity is the source- and receiver-chamber concentration history, not the diffusion coefficient itself. The coefficient is inferred through a transport and chamber-balance model. Unless leakage, air-side resistance, chamber mixing, detector dynamics, and pressure differences are measured or shown to be negligible, the fitted result should be reported as *D_app_* rather than as an intrinsic material property. Blank-holder tests, pressure-decay or tracer-leak tests, background runs, and replicate specimens are therefore part of the measurement method rather than optional quality checks.

### 3.2. Experimental Configurations

#### 3.2.1. Single Source-Receiver Configuration

**Figure 1:**
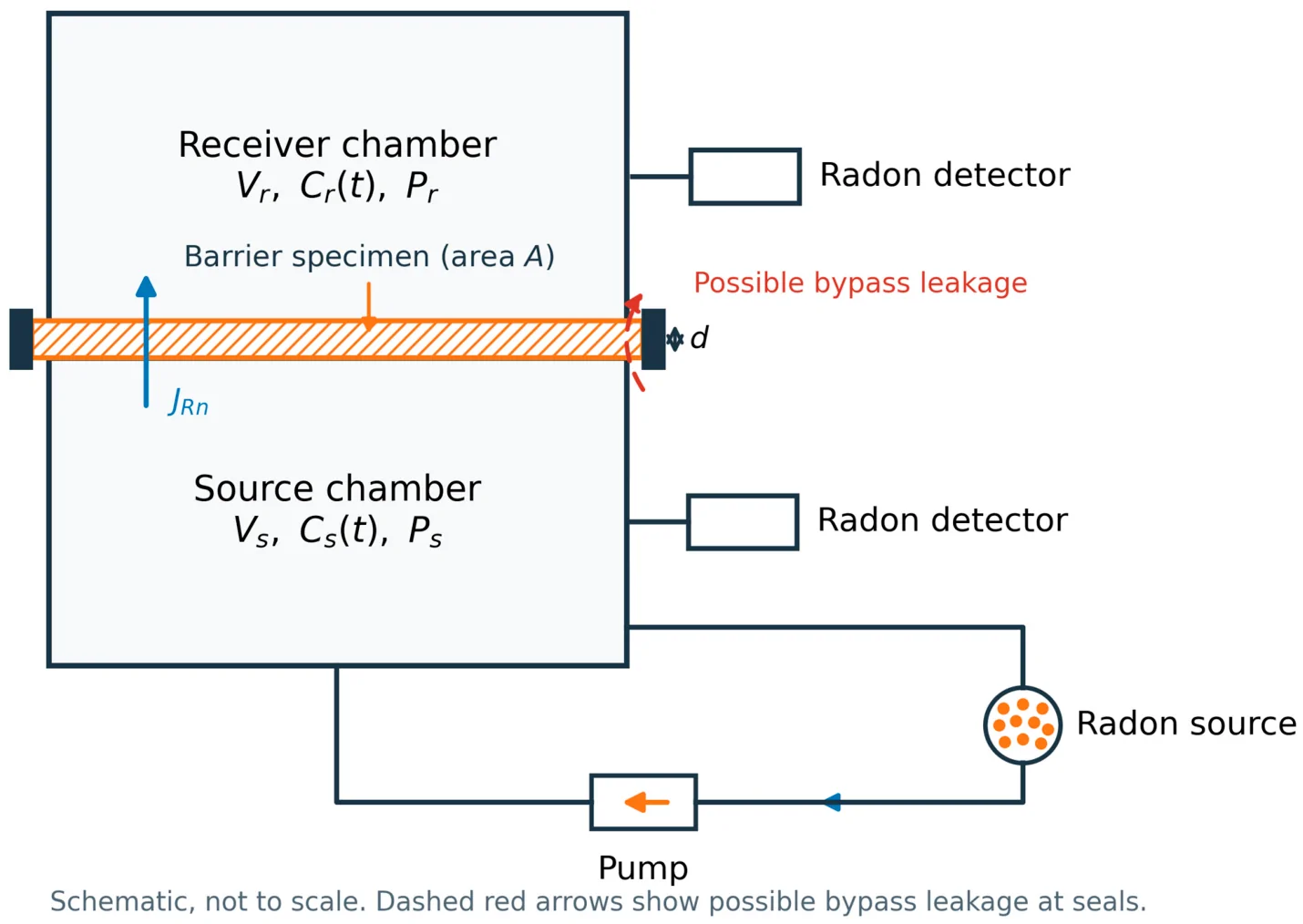
Schematic of a two-chamber system for measuring radon transport through a barrier specimen. The specimen has exposed area *A* and thickness *d*. Radon concentrations, chamber volumes, and pressures are represented by *C*, *V*, and *P*, respectively. The blue arrow indicates radon flux, and the dashed red arrow shows a possible bypass leakage path at the specimen seal. Adapted from [18]. Illustration prepared with the assistance of ChatGPT (OpenAI; GPT‑6 Astra Pro) and edited and verified by the authors.

A single source–receiver system separates two chambers by a test specimen [18] (Figure 1). It supports both transient and steady-state measurements. Steady state requires a time-independent profile; a linear concentration profile and spatially uniform flux are approximations valid only when decay within the specimen is negligible. Interpretation also requires defined partitioning, boundary transfer, and leakage.

#### 3.2.2. Dual Receiver Configuration

**Figure 2:**
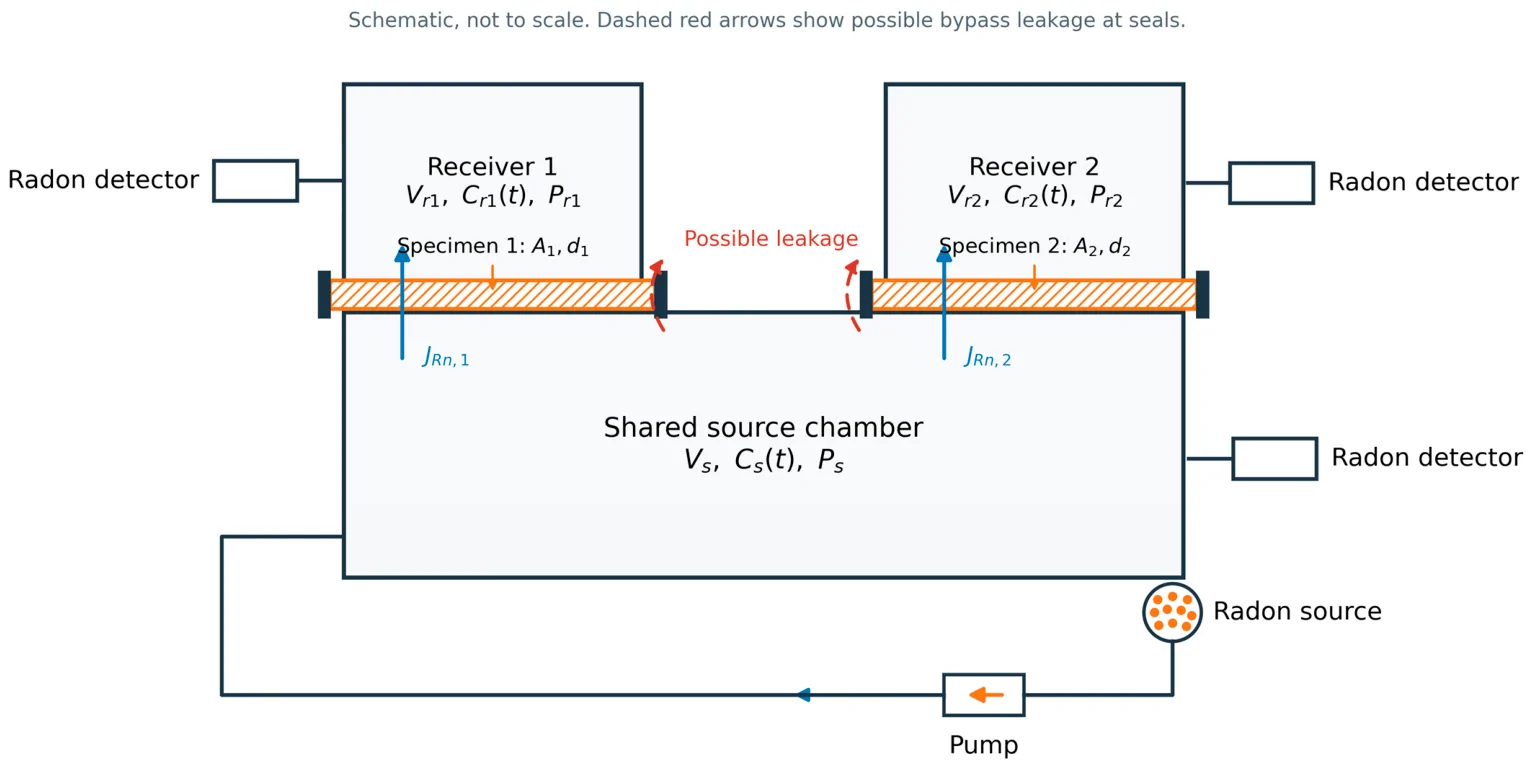
Schematic of a system for simultaneously testing two barrier specimens using one source chamber and two receiver chambers. Each specimen may have a different exposed area and thickness. Separate receiver detectors allow radon transport through each specimen to be evaluated. Dashed red arrows indicate possible leakage at the seals. Adapted from [37]. Illustration prepared with the assistance of ChatGPT (OpenAI; GPT‑6 Astra Pro) and edited and verified by the authors.

A central source chamber can supply two receiver chambers through separate specimens (Figure 2). This arrangement permits simultaneous comparison under a shared source history [37]. It can reduce the total time required for sequential tests, but it does not eliminate source fluctuations or differences between receiver conditions. Source concentration, receiver volumes, detector responses, and leakage must be included in the joint analysis.

#### 3.2.3. Parallel Double-Container Configuration

**Figure 3:**
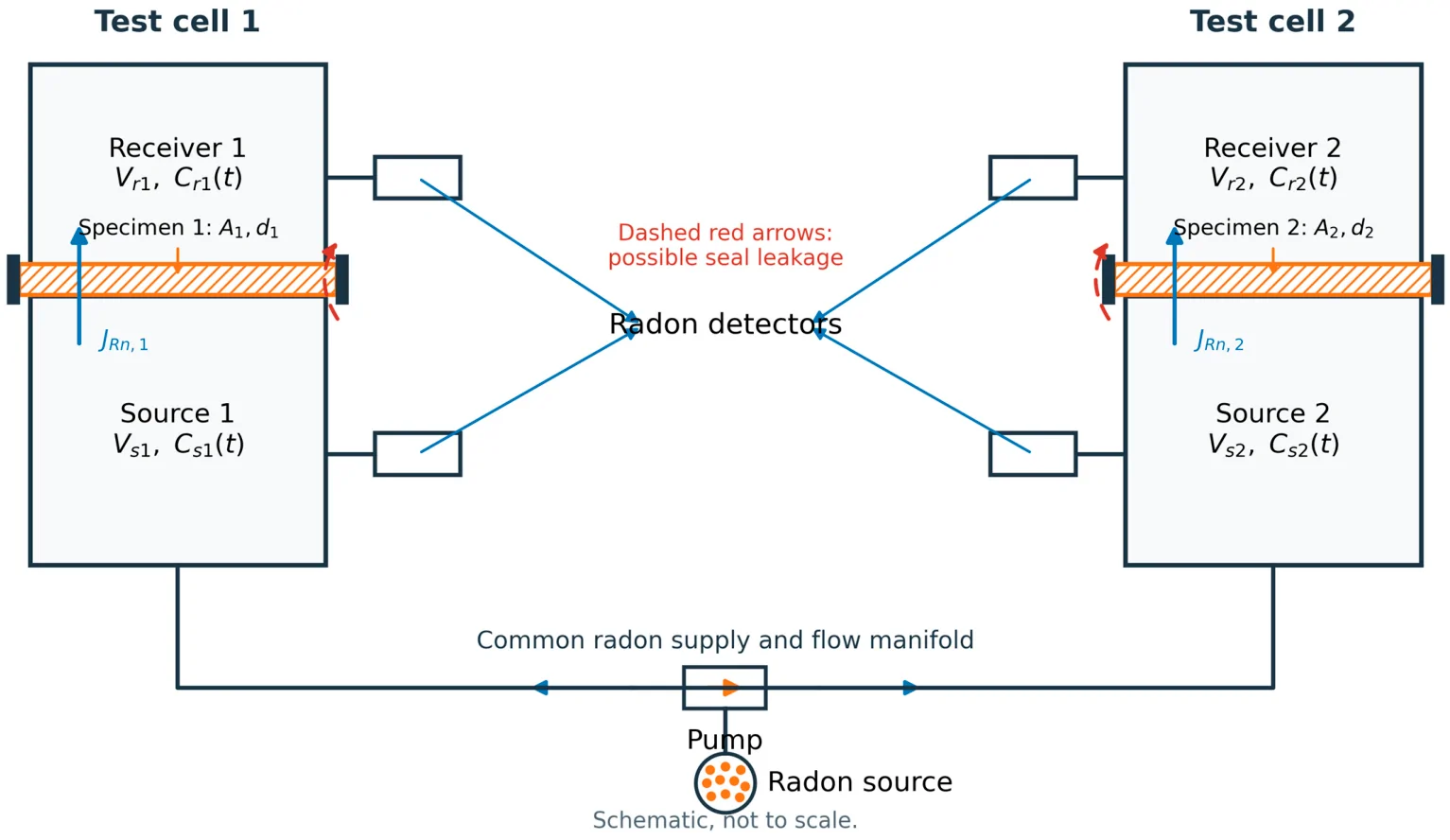
Conceptual parallel-cell arrangement with a common radon supply. The drawing is not a complete piping diagram: supply, return or exhaust, detector-loop connections, and pressure-control paths must be specified for the implemented apparatus. Arrows must not be interpreted as a complete constant-volume flow balance. Adapted from [9]. Illustration prepared with the assistance of ChatGPT (OpenAI; GPT‑6 Astra Pro) and edited and verified by the authors.

Parallel source–receiver cells can increase throughput and support comparisons across specimens or controlled environmental conditions [8,9,10]. A common supply does not guarantee identical source-side concentrations or pressures. Each branch therefore requires a defined flow path and measurement or modeling of its local source conditions. Figure 3 illustrates the intended comparison; its final plumbing details require confirmation before experimental reproduction.

Comparative appraisal. The three configurations trade throughput against control of the boundary conditions. The single source–receiver cell (Figure 1) has the simplest and most transparent mass balance and is the basis of ISO/TS 11665-13 [25]; its main weaknesses are low throughput and the absence of an internal check on seal leakage unless a blank-holder run is added. The dual-receiver arrangement (Figure 2) exposes two specimens to an identical source history, which is advantageous for paired comparisons (for example, an intact specimen and a specimen containing a joint), but it couples the two measurements through the source chamber, so that leakage or sorption in one branch alters the source concentration seen by the other. Parallel cells (Figure 3) maximize throughput and allow environmental factors to be varied, but they introduce the largest uncertainty in the local source concentration and pressure of each branch, and therefore the greatest risk that inter-cell differences are attributed to the materials rather than to the apparatus. For highly resistant membranes, where leakage-equivalent transfer may approach specimen transfer, the single cell with independent leakage characterization is generally preferable; for screening of more permeable materials, the higher throughput of multi-cell systems is usually the better trade-off.

#### 3.2.4. Boundary Conditions and Chamber Mass Balances

The governing equation is incomplete without initial and boundary conditions. For the outward unit normal **n**, the common boundary types are [14]:

Dirichlet condition (specified concentration):

*C*(**x**,*t*) = *C_b_*(*t*) (11)

Neumann condition (specified flux):

−*D*∇*C* ⋅ **n** = *J_b_*(*t*) (12)

with *J_b_* = 0 for an impermeable boundary.

Robin condition (finite air-side mass transfer):

(13) $$-D_{m}\nabla C_{m}\cdot n=h\;\left[\frac{C_{m}}{K}-C_{g,b}\right]$$

For Equation (13), *C*_m_ is the material-phase surface concentration, *C*_g,b_ is the bulk-gas concentration, *K* = *C*_m,s_/*C*_g,s_ is the equilibrium partition coefficient at the interface, *h* is a gas-side mass-transfer coefficient, and **n** is the outward unit normal. Both terms in the concentration difference are on a gas-phase basis [14,19,20]. If gas-side resistance is negligible, *C*_m,s_ = *KC*_g,b_; setting *C*_m,s_ = *C*_g,b_ additionally assumes *K* = 1. A prescribed Dirichlet value in Equation (11) must likewise be defined on the modeled phase basis. The conditions under which gas-side resistance may be neglected are quantified in Section 5.3.2.

For a well-mixed receiver chamber, a general activity balance can be written as

(14) $$V_{r}\frac{dC_{r}}{\mathrm{dt}}=AJ_{\mathrm{in}}+S_{\mathrm{bg},r}+Q\left(C_{\mathrm{in}}-C_{r}\right)+k_{l,r}V_{r}\left(C_{\mathrm{amb}}-C_{r}\right)-\lambda V_{r}C_{r}$$

here *V_r_* is the receiver volume, *A* is the exposed specimen area, *J*_in_ is inward specimen flux, *S*_bg,*r*_ is an activity source rate (Bq s^−1^), *Q* is balanced through-flow with equal inlet and outlet volume rates, *k*_𝓁,*r*_ is the ambient-exchange constant, *C*_amb_ is ambient activity concentration, and *λ* is the radioactive decay constant [15,16,17,24,25]. The leakage term permits both radon gain and loss.

A closed detector-recirculation loop returns sampled gas and does not constitute ventilation [11,16]. The *Q*(*C*_in_ − *C_r_*) term is absent for an ideal closed loop. Include detector and tubing volumes in the mixed volume or model them as separate compartments when mixing and transit delays matter. An analogous source balance includes radon production, decay, leakage, and specimen transfer. Simultaneous solution of the specimen and chamber equations provides a consistent transient model [15,16,17].

Fixture leakage constitutes a transport pathway operating in parallel with diffusion through the specimen. If *G_l_* is a calibrated leakage conductance in cubic metres per second, the total measured activity-transfer rate is

(15) $${\dot{R}}_{\mathrm{meas}}=AJ_{m}+G_{l}\left(C_{\mathrm{sc}}-C_{\mathrm{rc}}\right)$$

or, when expressed as flux,

(16) $$J_{\mathrm{meas}}=J_{m}+\frac{G_{l}}{A}\left(C_{\mathrm{sc}}-C_{\mathrm{rc}}\right)$$

The activity-transfer rate in Equation (15) has units of Bq s^−1^; the flux in Equation (16) has units of Bq m^−2^ s^−1^. *G_l_* describes concentration-driven inter-chamber exchange and differs from ambient exchange k_l_ and pressure-driven bypass flow. For one-way bypass flow, include the upstream inflow and compensating outflow explicitly. Leakage uncertainty must propagate to the fitted coefficient. Portaro et al. [17] evaluated background, holder leakage, and detection limits. Transfer unresolved above leakage and background should be reported as a detection-limit statement or a model-based upper bound.

Illustrative leakage example. The magnitude of leakage bias can be shown with a steady-state analysis that neglects decay within the specimen. If the measured transfer of Equation (16) is interpreted with Equation (18), the inferred coefficient becomes

(17) $$D_{\mathrm{app}}=D+\frac{G_{l}d}{A}=D+D_{\mathrm{leak}}$$

so that undetected inter-chamber leakage adds a leakage-equivalent diffusion coefficient *D*_leak_ = *G*_l_*d*/*A* that is independent of the source concentration. For a specimen of area *A* = 0.01 m^2^ (about 11 cm in diameter) and thickness *d* = 2 mm, a leakage conductance of only *G*_l_ = 10^−11^ m^3^ s^−1^ (an exchange of about 0.9 mL of gas per day) gives *D*_leak_ = 2 × 10^−12^ m^2^ s^−1^. Relative to the category means reported by Jiránek and Kotrbatá [38], this would bias *D*_app_ upward by about 1.5% for polymer–cement coatings (1.3 × 10^−10^ m^2^ s^−1^), by about 11% for PVC (1.8 × 10^−11^ m^2^ s^−1^), by about 33% for HDPE (6.1 × 10^−12^ m^2^ s^−1^), and by a factor of about 8 for aluminum-film bitumen membranes (2.9 × 10^−13^ m^2^ s^−1^). Because the diffusive conductance of a seal gap scales as *D*_0_ × (gap cross-section)/(gap length), a continuous peripheral gap only 1 µm wide along a 0.35 m perimeter and a 5 mm seal width would already give *G*_l_ ≈ 8 × 10^−10^ m^3^ s^−1^, nearly two orders of magnitude above this example; blank-holder and leak tests are therefore indispensable for highly resistant materials.

For membranes with *d* ≫ *L*_D_, radioactive decay attenuates the specimen flux (Section 6.4) but not the short leakage path, so the relative bias is even larger. The sign of the bias depends on the pathway: inter-chamber leakage inflates *D*_app_, whereas unmodeled loss from the receiver to laboratory air (*k*_l_ in Equation (14)) biases *D*_app_ downward; in the TESTMAT system this loss was about 7% of *λ* [17]. A practical criterion is to demonstrate by blank-holder tests that *D*_leak_ is below about 10% of the expected coefficient, or otherwise to determine *G*_l_ independently and include it in the fitted model.

#### 3.2.5. Detector Response, Calibration, and Background

Detector characteristics matter here only insofar as they affect the inferred coefficient. When transient concentration histories are fitted, detector dynamics become part of the inverse problem: a response time that is not short compared with the breakthrough time distorts the curve shape and, if unmodeled, is absorbed into *D*_app_ [16,17]. Detector calibration, background correction, and leakage characterization must be adequate to ensure that the measured concentration histories support reliable estimation of the diffusion coefficient. Calibration should be traceable to a recognized radon activity standard, and the instrument model, operating mode, cycle time, and calibration certificate should be reported. Table 1 summarizes the detector-related issues that most often affect the result.

### 3.3. Steady-State Measurement Methods

#### Classical Steady-State Approach

At steady state, and only when decay within a thin specimen is negligible, one-dimensional transport may be approximated [39] by

(18) $$J=D_{\mathrm{app}}\frac{\Delta C}{d}$$

This expression is a flux-gradient relation, not a complete test method. It assumes a linear concentration profile, specified interface concentrations, constant *D*, no air-side resistance, no partition discontinuity, and no leakage or advection. If gas-phase chamber concentrations are used for a dense polymer, the inferred coefficient can include the partition coefficient and is more properly interpreted as a permeability-related or apparent quantity. When *d/L_d_* is not small, radioactive decay produces a curved profile and the full solution in Section 4.1 is required.

The main advantage of a steady-state analysis is transparency: it uses a time-independent flux and avoids estimating an unknown initial concentration profile. Its disadvantages are potentially long test duration, susceptibility to drift and leakage, and poor identifiability for very resistant membranes. Steady state should be demonstrated from a pre-defined slope or flux criterion rather than assumed after a fixed elapsed time.

Critical appraisal. Steady-state analysis is attractive because the measurand follows directly from a measured flux and concentration difference, but its reliability depends on whether steady state has actually been reached. For highly resistant membranes with *d* ≫ *L*_D_, the slowest relaxation time approaches the ^222^Rn mean life (about 5.5 d), so that two to three weeks are needed, during which source drift, background change, and leakage accumulate [15,39]. The first international comparison of radon diffusion-coefficient measurements was organized to quantify such inter-laboratory differences [40], and the subsequent review of 16 methods found that results for identical membranes could differ by up to two orders of magnitude, mainly because tests were too short, source concentrations were too low, and steady-state equations were applied to data obtained before steady state [41]. The steady-state approach is therefore best suited to materials with comparatively high coefficients, such as porous building materials and permeable coatings, for which equilibrium is reached within hours to days and the specimen flux is large relative to leakage.

### 3.4. Transient Measurement Methods

#### 3.4.1. Instantaneous Exposure Method

For a concentration signal that follows a single exponential, an empirical rate constant may be calculated as:

(19) $$K_{\mathrm{obs}}=\frac{1}{t}\ln\left[\frac{C_{0}}{C\left(t\right)}\right]$$

Here *C*_0_ = *C*(0), *t* > 0, and *K*_obs_ is a rate constant with units of s^−1^. The expression assumes a zero asymptote and positive concentration. A nonzero background or asymptote requires a corresponding offset model.

Equation (19) does not determine a diffusion coefficient. The conversion from a measured decay rate to *D* depends on cell volume, membrane area, thickness, partitioning, radioactive decay, leakage, and boundary conditions. A modified-cell method requires its own validated transport and chamber balance; no universal conversion D = d^2^K_obs_ is assumed here.

#### 3.4.2. Accumulation Method

Accumulation methods monitor radon entering a receiver chamber [15,16,17,24]. When a constant asymptote and one dominant first-order mode provide an adequate approximation, the receiver signal can be represented by

(20) $$C_{r}\left(t\right)=C_{r,\infty }+\left(C_{r}(0)-C_{r,\infty }\right)e^{-k_{\mathrm{eff}}t}$$

Here *C*_r,∞_ is the empirical asymptote and *k*_eff_ is the fitted first-order rate constant. Equation (20) includes the measured initial concentration. It is not the general exact solution of a coupled membrane–chamber system, and *k*_eff_ does not uniquely identify *D*. Material-property inference requires the complete transport and measurement model.

#### 3.4.3. Transient Numerical Fitting

Analytical and numerical models can both include radioactive decay. Numerical solutions provide flexibility for complex geometries, variable properties, and coupled chamber or boundary conditions. Their use does not by itself shorten measurements or improve accuracy. Parameter estimation and uncertainty propagation must account for initial conditions, leakage, detector response, and model adequacy [15,16,17].

Critical appraisal. Transient methods shorten the test and use the complete concentration history, which is decisive for highly resistant membranes whose steady state would take weeks [15,17]. Their weakness is the converse of their strength: because the coefficient is inferred from the shape of the curve, any unmodeled process that also shapes the curve (initial specimen loading, detector lag, chamber mixing, leakage, or sorption) is absorbed into *D*_app_. The instantaneous-exposure approach (Section 3.4.1) is fast but yields a lumped rate constant whose conversion to *D* requires a validated cell model [42]; the accumulation approach is robust for moderate coefficients, but its single-exponential form (Equation (20)) becomes ambiguous when more than one time constant is present; and full numerical inversion is the most general but is only as reliable as its forward model, and requires explicit identifiability checks (Section 4.4). Table 2 summarizes these trade-offs.

### 3.5. Recent Advances in Measurement Systems

#### 3.5.1. TESTMAT Small-Chamber System

Portaro et al. [17] introduced TESTMAT, a compact PVC source-receiver apparatus arranged according to the two-sided ISO geometry. The ENDORSE code solves the transient diffusion-decay problem with an explicit finite-difference scheme and uses Monte Carlo propagation to estimate uncertainty. The study also measured background evolution, evaluated a sample holder, and reported a leakage constant of approximately 7% of the ^222^Rn decay constant. The small receiver volume improves accumulation sensitivity, and the weak source reduces source-handling requirements.

Compared with conventional ISO-type cells, the principal advance of TESTMAT lies less in the apparatus than in the integration of measured background, quantified holder leakage, and Monte Carlo propagation into the inversion. Its limitations are those of any compact cell: the small exposed area increases the relative influence of edge sealing, and the leakage-equivalent coefficient *G*_l_*d*/*A* (Equation (17)) increases as *A* decreases. Replication on the same membranes in independent laboratories would establish whether the approach reduces inter-laboratory scatter.

#### 3.5.2. Multi-Chamber and Two-Loop Systems

Multi-chamber and two-loop configurations use separate recirculation circuits to follow both source and receiver concentrations, and numerical analysis of such systems has been used to predict equilibration times and interpret exchange rates [16]. Their advantage over single-detector cells is that the source history is measured rather than assumed. The cost is a more complex measurement model: tubing and detector volumes, circulation delays, loop leakage, and pump-induced pressure differences must be included, and a recirculation flow must not be treated as chamber ventilation unless gas is added or removed. These systems are most valuable as benchmarks for validating numerical models rather than as routine test devices.

#### 3.5.3. Room-Scale Physical Analogue Experiments

Scale-model rooms containing radon-emitting porous media provide a bridge between coupon measurements and building behavior [8,43]. They permit controlled assessment of ventilation, pressure, sealing, and barrier configuration.

Scale-model results show that measured reduction depends on membrane type, pressure regime, ventilation, and sealing [43]. Pressurization improved performance in some configurations, but a scale-model percentage is not a transferable field efficiency. These experiments are best used to test coupled mechanisms and to validate building models before occupied-building studies.

Scale-model rooms thus occupy an intermediate validation level between coupon cells and occupied buildings [8,44]: the observed percentage reduction is specific to the model geometry, source, leakage distribution, and operating conditions, and extrapolation to buildings requires a validated mass-balance model with explicit uncertainty for installation defects.

#### 3.5.4. Recent ISO-Based and Validation Studies

Recent work has broadened the evidence base beyond classical steady-state tests. Tejado-Ramos et al. [45] proposed a membrane assessment method for construction and refurbishment applications. Castaño-Casco et al. [46] focused on a more robust methodology across different material classes. Portaro et al. [17] combined an ISO-type two-chamber cell with transient numerical inversion, background characterization, leakage testing, and Monte Carlo propagation. Jiránek et al. [27] examined how membrane joints alter whole-system resistance, reinforcing the need to separate coupon properties from assembly performance. At a larger scale, Portaro et al. [43] tested airtight membranes in a scale-model room with pressurization.

These studies are complementary rather than interchangeable. ISO-type cells estimate a specimen parameter under controlled boundary conditions; joint tests quantify an assembly; and room or field studies measure the combined effects of barriers, pressure, ventilation, and workmanship. Long-term occupied-building studies rarely allow *D_m_* or *D_eff_* to be identified uniquely. Their proper role is therefore validation of predicted building performance, not replacement of a controlled material measurement.

Critical appraisal of data scatter. The studies above differ in specimen fabrication (factory sheet versus laboratory-cast coating, conditioning history), apparatus (chamber volumes, exposed area, source strength), and data analysis (steady-state flux versus transient inversion, with or without leakage and detector terms). These protocol differences, rather than differences between materials, are a major source of scatter in the literature. Three observations support this conclusion. First, identical membranes measured by different methods have differed by up to two orders of magnitude [41]. Second, the coefficients reported for very thin or metallized foils, about 1 × 10^−13^ m^2^ s^−1^ for 15 µm aluminum foil and 10^−14^–10^−13^ m^2^ s^−1^ for polyethylene–aluminum multilayers [34], are difficult to reconcile with the expected impermeability of intact aluminum to noble gases; they are better interpreted as apparent values limited by pinholes, edge sealing, or the detection capability of the apparatus. Third, a small number of pinholes can change the measured coefficient materially [35]. A coefficient reported without its measurand definition, boundary conditions, leakage verification, and conditioning history therefore cannot be placed reliably on a common scale with other values.

## 4. Numerical Modeling Approaches

### 4.1. Steady-State Analytical Solutions

For a homogeneous slab with constant D and no internal source or advection, let *C*_s,u_ and *C*_s,d_ denote the two material-phase surface concentrations. The steady profile is [14]

(21) $$C\left(x\right)=\frac{C_{s,u}\sinh\left(\kappa \left(d-x\right)\right)+C_{s,d}\sinh\left(\mathrm{\kappa x}\right)}{\sinh\left(\mathrm{\kappa d}\right)}$$

The receiver-face activity flux, positive in the +x direction, is

(22) $$J_{r}=-D{\left.\frac{\mathrm{dC}}{\mathrm{dx}}\right|}_{x=d}=\mathrm{D\kappa}\frac{C_{s,u}-C_{s,d}\cosh\left(\mathrm{\kappa d}\right)}{\sinh\left(\mathrm{\kappa d}\right)},$$

(23) $$\kappa =\sqrt{\frac{\lambda }{D}}$$

For the same convention, $J_{s}=\frac{\mathrm{D\kappa}\left[C_{s,u}\cosh\left(\mathrm{\kappa d}\right)-C_{s,d}\right]}{\sinh\left(\mathrm{\kappa d}\right)}$. For a dense membrane, use *D* = *D*_m_, *C*_s,u_ = *K C*_g,u_, and *C*_s,d_ = *K C*_g,d_. The factor *K* then appears in both gas-concentration-based fluxes. The difference between face fluxes equals decay within the slab. These expressions provide analytical benchmarks for numerical models.

### 4.2. Numerical Solution of the Forward Model

Transient problems with time-dependent chamber concentrations, layered or heterogeneous specimens, joints, or multidimensional geometry require numerical solution. Finite-difference and finite-volume schemes are efficient for one-dimensional slabs coupled to chamber balances [16,17,47,48], whereas finite-element methods are better suited to two- and three-dimensional geometries with material discontinuities, joints, penetrations, and thin membrane layers [26,49]. Computational fluid dynamics (CFD) extends these models to coupled diffusion and convection in rooms, wall cavities, and sub-slab regions, typically by solving a convection–diffusion equation for radon in a separately computed flow field [50]. Treating radon as a passive scalar is justified at trace concentrations, whereas CFD studies of tracer transport show that tracers which alter the fluid density require a coupled treatment [51].

The choice of method is less important for the inferred coefficient than its verification. Explicit time integration requires a stability restriction on the time step (and, for concentrations, a stricter positivity restriction), whereas implicit schemes are unconditionally stable but can still produce non-physical oscillations near sharp gradients [47,48]; when advection is significant, stabilized formulations such as the streamline-upwind/Petrov–Galerkin method should be used [32,33]. In every case, the reported model should specify the spatial grid, time step, and boundary-node treatment, and should demonstrate grid and time-step convergence, a global radon mass balance (inventory change equal to inputs minus decay, leakage, and outputs) within a stated tolerance, and agreement with the analytical solutions of Section 4.1 in limiting cases [47,52].

### 4.3. Explicit Modeling of Joints and Leaks

Recent work has given increased attention to non-ideal transport pathways, including membrane joints, seams, penetrations, and leakage around the specimen holder [17,27]. Earlier experimental and numerical studies also demonstrated that cracks, structural joints, and pipe penetrations can substantially increase radon entry [26,53]. In numerical simulations of complete barrier assemblies, transport through joints may make a larger contribution to the total radon flux than diffusion through the intact membrane, particularly when the joint is poorly sealed [26,27]. Under these conditions, the diffusion coefficient of the intact material alone is insufficient to predict the performance of the installed barrier system.

### 4.4. Inverse Modeling, Parameter Estimation, and Uncertainty

Inverse modeling estimates transport parameters by fitting the solution of the diffusion–decay equation and the coupled chamber mass-balance equations to the measured concentration–time data [16,17]. More generally, this procedure is an inverse problem for a system governed by partial and ordinary differential equations [54]. The estimated quantity is an apparent diffusion coefficient unless the forward model adequately represents boundary transfer, chamber mixing, detector response, background, leakage, and any other relevant transport pathway.

Parameters are usually estimated by weighted or generalized least squares, minimizing the covariance-weighted residuals between measured and simulated chamber concentrations [16,17,55]. Estimating ln *D* enforces positivity and improves scaling. Identifiability should be assessed through sensitivity analysis, parameter correlations, or profile likelihoods; where *D*, *K*, leakage, and detector response cannot be separated, the identifiable composite parameter should be reported instead.

Uncertainty evaluation should follow the GUM (JCGM 100) [56], beginning with an explicit definition of the measurand, for example “the apparent ^222^Rn diffusion coefficient of a 0.25 mm membrane at 23 °C and 30% relative humidity, determined with the stated two-chamber transient model”. Because the inverse problem is nonlinear and the output distribution is often skewed, Monte Carlo propagation according to JCGM 101 [57], with each realization repeating the complete fit, is generally preferable to linearization, as applied to radon membranes by Portaro et al. [17]. Bayesian inference is a complementary option when independently justified prior information exists, but its benefit for radon-barrier measurements remains to be demonstrated [58,59]. Results should state the coverage factor or coverage probability, or the coverage-interval endpoints for skewed distributions.

Model-form uncertainty. Most published studies report only the statistical standard deviation of the fitted coefficient, which is conditional on the chosen model. This omits model-form uncertainty, the error caused by an inadequate transport, boundary, or chamber model [60], which can exceed the statistical component by a wide margin: the differences of up to two orders of magnitude found for identical membranes [41] are far larger than any reported counting uncertainty and arise mainly from model and protocol choices. A practical assessment is a multi-model comparison in which the same data are fitted with a set of plausible alternatives, for example Dirichlet versus Robin interfaces, with versus without inter-chamber leakage and receiver loss, a one-compartment versus a two-compartment chamber with detector lag, and a one-dimensional versus an edge-corrected geometry. Models rejected by residual diagnostics or by independent data (blank-holder tests, alternative thicknesses) are discarded. The spread of the coefficients from the remaining models provides an estimate of the model-form component; if its half-range *a* is treated as a rectangular distribution, *u*_model_ = *a*/√3, and, assuming independence, the combined standard uncertainty is *u*_c_^2^ = *u*_param_^2^ + *u*_model_^2^. Alternatively, information-criterion weights or Bayesian model averaging can be used to combine the candidate models [61]. The most convincing evidence of model adequacy is nevertheless experimental: consistency of the coefficient across specimen thicknesses, source concentrations, and apparatus, because model-form errors generally do not scale in the same way as true material transport.

## 5. Factors Influencing Radon Diffusion Coefficients

A radon diffusion coefficient should not be treated as a single invariant quantity independent of material state and measurement method. At specified temperature, composition, and physical state, the molecular diffusion coefficient *D_m_* describes radon mobility within a homogeneous phase. In porous or heterogeneous materials, the effective coefficient *D_eff_* additionally depends on connected porosity, tortuosity, constrictivity, and moisture distribution. An experimentally fitted apparent coefficient *D_app_* may also depend on specimen preparation, boundary conditions, leakage, detector response, and the assumptions of the inversion model. The coefficient type and test conditions must therefore accompany every reported value [19,25,38,62,63].

Table 3 identifies, for each influencing factor discussed below, which of the three coefficients is physically modified and which is affected only through the measurement or inversion. The distinction matters because a factor that alters *D*_app_ without altering *D*_m_ or *D*_eff_ (for example, leakage or detector lag) is a measurement artifact to be corrected, whereas a factor that alters *D*_m_ or *D*_eff_ (for example, temperature or moisture) is a genuine change in transport that must be specified as a test condition.

### 5.1. Material Properties

#### 5.1.1. Porosity and Pore Structure

In porous media, the effective diffusion coefficient is controlled primarily by the connected gas-filled pore space rather than total porosity alone. A general representation is

*D*_eff_ = *D*_a_*F*(*ε*_g_, *τ*, *χ*) (24)

where *D*_a_ is the diffusion coefficient in free air, *ε*_g_ is gas-filled porosity, *τ* represents tortuosity, and *χ* represents constrictivity. Expressions such as *D*_eff_ = *D*_a_*ε*_g_*χ*/*τ* are simplified constitutive relations, and the precise definitions of tortuosity and constrictivity must be stated [22,23,63].

Dry, coarse materials with well-connected pores may exhibit effective coefficients of the order of 10^−6^–10^−5^ m^2^s^−1^, approaching a fraction of the free-air value. Materials containing disconnected pores, such as closed-cell foams, can have much lower coefficients despite having appreciable total porosity. In waterproofing products, experimentally determined coefficients span approximately 10^−15^–10^−8^ m^2^s^−1^, although commonly used polyethylene, polypropylene, flexible polyvinyl chloride, and bituminous membranes generally fall within a considerably narrower range of approximately 3 × 10^−12^–3 × 10^−11^ m^2^s^−1^ [38]. These values describe manufactured products under specified test conditions and should not be interpreted as universal intrinsic coefficients for the corresponding pure polymers.

Microstructure-based effective-property models developed for other transport coefficients of cementitious materials, such as the thermal conductivity of concrete [64], follow the same effective-medium logic and illustrate how porosity, pore-size distribution, and phase connectivity control a macroscopic coefficient; analogous microstructural models for *D*_eff_ require validation with radon-specific measurements.

#### 5.1.2. Material Composition

Radon transport through dense polymers is commonly described by a solution–diffusion process comprising sorption at the upstream surface, molecular diffusion through the polymer, and desorption at the downstream surface. The material diffusion coefficient D_m_ describes mobility within the polymer, whereas the partition coefficient K describes equilibrium between the polymer and gas phases. Under a concentration-based convention, the permeability is

P = KD_m_ (25)

Polymer density, crystallinity, free volume, additives, and manufacturing history affect diffusion and partitioning. The multi-product survey in [38] reported a lower mean coefficient for HDPE than LDPE, with overlapping ranges; this does not establish a universal ranking of products. Separate diffusion and solubility measurements [20] and temperature-dependent polymer measurements [19] illustrate the need to define both quantities.

### 5.2. Environmental Factors

#### 5.2.1. Temperature

For a specified material and temperature interval, the temperature dependence of molecular diffusion is often represented by an Arrhenius relation:

(26) $$D_{m}\left(T\right)=D_{\mathrm{pre}}\exp\left(-\frac{E_{D}}{\mathrm{RT}}\right)$$

Here *D_pre_* is the pre-exponential factor (distinct from free-air diffusivity D_0_), *E_D_* is an apparent activation energy, R is the molar gas constant, and T is absolute temperature. Parameters are specific to the material and fit temperature range [65]. Polymer measurements demonstrate temperature-dependent diffusion and partitioning [19]; they do not justify a universal activation energy.

Temperature therefore modifies *D*_m_ and *K*, and hence *P*, as genuine material properties; in porous media it affects *D*_eff_ mainly through the weak temperature dependence of gas-phase diffusivity and through moisture redistribution (Table 3).

#### 5.2.2. Moisture Content

Moisture affects radon generation and transport through different mechanisms that should not be conflated. Moderate moisture can increase the emanation coefficient by enhancing the release of radon atoms from mineral grains, whereas increasing water saturation generally reduces gas-phase diffusion by decreasing connected air-filled porosity and increasing pathway tortuosity [62,63,66].

Radon also partitions between the gas and aqueous phases:

*C*_w_ = *K*_wg_(*T*)*C*_g_, (27)

where *K*_wg_ is the water-to-air partition coefficient. Its value is approximately 0.25–0.26 in freshwater near 20 °C, but it depends on temperature and salinity [67]. Consequently, no universal pair of “dry” and “saturated” diffusion coefficients should be assigned to all porous materials. Measurements and predictive correlations demonstrate that increasing water saturation can reduce *D*_eff_ by one or more orders of magnitude, but the magnitude depends on porosity, pore-size distribution, particle size, and saturation history [63,66]. In polymer membranes, water uptake may alter sorption and chain mobility; this effect should be measured for each formulation rather than assumed negligible.

For meaningful comparison, the moisture state should be reported as volumetric water content or degree of saturation together with the conditioning relative humidity and duration, not merely as “dry” or “wet” (Section 6.2). The same air–water partitioning underlies the metrology of radon in water, where established degassing and emanation techniques and their calibration [12,68] provide a useful reference for the treatment of aqueous radon in moist building materials.

#### 5.2.3. Pressure Gradients

Diffusion is driven by concentration gradients, whereas pressure differences generate bulk gas flow. A simplified total flux may be written as

J*_g_* = −D_bulk_∇C*_g_* + u*_D_*C*_g_* (28)

In Equation (28), *u*_D_ is the Darcy velocity and *D*_bulk_ includes gas-filled porosity; pore-gas velocity instead pairs with *D*_pore_. Pressure differences can drive entry through cracks, joints, and penetrations [6,29,30,31]. The consistent Peclet number is that of Equation (9), and an order-of-magnitude example is given in Section 2.4. A pressure difference does not alter *D*_m_ or *D*_eff_, but unmodeled advection is absorbed into *D*_app_ (Table 3).

### 5.3. Sample Preparation and Testing Conditions

#### 5.3.1. Sample Thickness

Specimen thickness affects signal magnitude, measurement duration, and parameter identifiability. The relation J ∝ 1/d assumes negligible decay and fixed surface concentrations. When d is comparable to the diffusion length, use the full diffusion–decay solution with the stated boundary concentrations [14,69]. Thickness does not change *D*_m_ or *D*_eff_ of a homogeneous material; it affects *D*_app_ through decay attenuation, parameter identifiability, and the relative weight of defects (Table 3).

There is no universal optimum thickness range applicable to all material classes. Thickness should be selected through a preliminary sensitivity analysis using the expected coefficient, partition coefficient, chamber volumes, detector sensitivity, leakage-equivalent detection limit, and planned measurement duration [15,25,39,69]. The actual mean thickness and its spatial variation should be reported. ISO/TS 11665-13:2025 [25] applies to waterproofing materials and should not be cited as prescribing representative thicknesses for soil or concrete.

#### 5.3.2. Edge Effects and Boundary Layers

One-dimensional transport requires a specimen with sufficiently large lateral dimensions relative to its thickness and an effective seal around the perimeter. Imperfect edge sealing creates a parallel bypass path, whereas finite specimen dimensions can produce multidimensional concentration gradients. Both effects can bias the fitted apparent diffusion coefficient, particularly when the specimen flux is close to the fixture-leakage background [16,17,24,25,26].

Finite gas-side transfer may be represented by the Robin condition

*J_inward_* = *h*(*C*_g,b_ − *C*_g,s_) = *h*(*C*_g,b_ − *C*_m,s_/*K*) (29)

For a dense membrane with negligible decay across the film, a one-sided mass-transfer Biot number is

(30) $${\mathrm{Bi}}_{m}=\frac{\mathrm{hd}}{P}\;$$

For negligible membrane decay, the complete series resistance is R_total_ = 1/h_u_ + d/P + 1/h_d_. Both gas-side resistances must be small relative to d/P before they can be neglected. If decay is appreciable, use the full diffusion–decay solution with both Robin conditions instead of a resistance-only approximation.

Engineering interpretation of *Bi*_m_. For equal transfer coefficients on both faces, the fraction of the total resistance contributed by the two gas-side films is 2/(*Bi*_m_ + 2) (for porous materials, *P* is replaced by *D*_bulk_). Gas-side resistance can therefore be neglected, contributing less than about 1%, when *Bi*_m_ ≳ 200, and it must be included explicitly when *Bi*_m_ ≲ 20, where it contributes about 10% or more. The transfer coefficient can be estimated as *h* ≈ *D*_0_/*δ*, where *δ* is the effective thickness of the concentration boundary layer: about 10^−2^ m s^−1^ for a well-stirred chamber (*δ* ≈ 1 mm) and of the order of 10^−4^ m s^−1^ for an unstirred chamber in which transfer relies on molecular diffusion over several centimeters.

Three practical cases follow: (i) Dense polymer membranes: with *P*/*d* ≈ 10^−8^ m s^−1^ (for example, *P* ≈ 10^−11^ m^2^ s^−1^ and *d* = 1 mm), *Bi*_m_ ≥ 10^4^ even in an unstirred chamber, so gas-side resistance is negligible and Dirichlet conditions are justified. (ii) Porous materials and permeable coatings: for *D*_bulk_ = 10^−7^ m^2^ s^−1^ and *d* = 10 mm, *P*/*d* = 10^−5^ m s^−1^ and *Bi*_m_ ≈ 10 in an unstirred chamber, so the gas-side films contribute about 17% of the total resistance and must be modeled, unless the chamber is mixed (*Bi*_m_ ≈ 10^3^). (iii) Highly permeable materials, such as open-cell insulation or thin porous layers with *P*/*d* approaching 10^−4^ m s^−1^: *Bi*_m_ approaches unity without forced mixing, and the measurement characterizes the chamber boundary layer as much as the material. Gas-side resistance should therefore be checked whenever porous or highly permeable materials are tested, whenever chamber mixing is not forced or not documented, and whenever thoron is measured, because its short diffusion length steepens near-surface concentration gradients.

#### 5.3.3. Aging and Degradation

Long-term barrier performance depends on the intact material, joints, penetrations, and installation quality. Relevant degradation mechanisms include changes in crystallinity and free volume, oxidation, plasticizer loss, ultraviolet exposure before covering, chemical attack, and repeated thermal or mechanical loading [70,71,72,73]. Their net effect on the molecular diffusion coefficient is material-specific: physical aging can reduce free volume, whereas embrittlement and cracking can create faster transport paths.

Construction-related punctures, tears, poorly sealed penetrations, and loss of joint adhesion can dominate total radon transmission even when the undamaged membrane has a very low diffusion coefficient [27,39]. Accordingly, aging studies should distinguish changes in the intact membrane coefficient from changes in seam conductance and defect-related leakage.

## 6. Reported Radon Diffusion Coefficients

Reported coefficients span several orders of magnitude, but comparisons require the measurand, concentration phase, geometry, environmental conditions, leakage treatment, and fitting model. Table 4 gives examples of reported coefficients together with the experimental conditions under which they were obtained. These datasets were acquired with different specimen thicknesses, temperature and humidity conditions, sample-preparation procedures, apparatus, and analysis models, and the resulting scatter is substantial. Direct numerical comparison of raw coefficient values from different studies therefore has limited practical meaning: a factor-of-two difference between two products tested in different laboratories cannot be attributed to the materials unless the measurand, boundary conditions, and leakage treatment are shown to be equivalent, and inter-method differences of up to two orders of magnitude have been reported for identical membranes [41]. The values in Table 4 should be read as indicating orders of magnitude and within-study rankings, not as a pooled database. Table 5, Table 6 and Table 7 identify the additional information needed for defensible comparisons; they are reporting checklists, not pooled estimates of material performance.

### 6.1. Dense Membranes and Thin Films

For dense polymers, transport is normally described as dissolution into the polymer followed by molecular diffusion [19,20]. Both the material diffusion coefficient *D_m_* and the dimensionless gas–polymer partition coefficient *K*, or their concentration-based product *P* = *KD_m_*, are required to predict flux from gas-phase concentrations. Reported membrane values are particularly sensitive to polymer grade, crystallinity, reinforcement, additives, thickness, temperature, and conditioning history [19,20,39]. ISO-based experimental investigations [11,17,45], together with the current ISO/TS 11665-13:2025 methodology [25], have improved the description of apparatus, boundary conditions, leakage testing, and transient analysis. Nevertheless, the resulting values remain method-dependent when partitioning, detector response, and leakage are treated differently. The minimum information needed to compare dense-membrane results is summarized in Table 5.

### 6.2. Porous Building Materials

Concrete, mortar, brick, gypsum, and insulation require an effective or multiphase transport description. Connected gas-filled porosity, tortuosity, constrictivity, moisture, and cracking can change *D_eff_* substantially [6,21,22,23,63,65,66,74]. Moisture commonly reduces gas-phase diffusion by interrupting connected gas-filled pathways, although the relationship is material-specific and may also affect partitioning and emanation [63,66,74]. Values measured using intact, conditioned specimens should not be transferred to cracked slabs without explicitly modeling joints or fractures [26,27]. The corresponding information for porous materials is listed in Table 6.

### 6.3. Coatings and Barrier Assemblies

Liquid-applied coatings and laminated systems can reduce surface radon flux, but their performance depends on cured thickness, substrate adhesion, overlaps, pinholes, penetrations, and aging [35,75,76,77,78]. Even a small number of pinholes can materially alter the measured apparent diffusion coefficient [35]. A coating tested together with its substrate must therefore be treated as an assembly rather than as an isolated material. When radioactive decay is negligible, multilayer resistances may be combined only if the concentration and partition conventions are consistent at every interface [14,34]. Long-term interpretation should additionally consider thermal, mechanical, chemical, and moisture-related degradation [70,71,72,73,79]. The information needed to compare coatings and barrier assemblies is listed in Table 7.

### 6.4. Thickness, Permeability, and Performance Metrics

For a dense membrane of thickness d, assuming negligible radioactive decay within the membrane and equilibrium partitioning,

(31) $$J=\frac{KD_{m}}{d}\left(C_{g,u}-C_{g,d}\right)=\frac{P}{d}\left(C_{g,u}-C_{g,d}\right)$$

Here, *C_g_*_,*u*_ and *C_g_*_,*d*_ are the upstream and downstream gas-phase activity concentrations. Under this concentration-based definition, the area-normalized membrane resistance is

(32) $$R_{m}=\frac{d}{P}=\frac{d}{KD_{m}}$$

This concentration-based permeability has units of m^2^ s^−1^ and differs from pressure-based gas-permeability conventions. When radioactive decay within the membrane is appreciable, the steady-state solution of Section 4.1 applies with *D* = *D*_m_ and *L*_D_ = (*D*_m_/*λ*)^1/2^. For a perfect sink at the receiver face (*C*_m,d_ = 0), the ratio of the receiver-face flux *J*_d_ to the corresponding no-decay flux, *J*_0_ = *D*_m_*C*_m,u_/*d*, is the decay-attenuation factor

(33) $$A_{\mathrm{decay}}=\frac{J_{d}}{J_{0}}=\frac{d/L_{D}}{\sinh\left(d/L_{D}\right)}\;$$

The sink-boundary condition must be stated; otherwise, this simplified attenuation factor is not valid.

*A_decay_* is a material-scale flux attenuation factor, not a whole-building radon reduction factor. Building-level performance additionally depends on soil-gas concentration, pressure-driven flow, joints and penetrations, ventilation, room volume, and internal source terms [27,30,31,44,63,80]. Laboratory diffusion coefficients, permeability, radon flux transmission, and measured indoor-concentration reduction should therefore be reported as distinct performance metrics.

### 6.5. Why Small Defects Can Dominate Radon Entry

A simple parallel-pathway estimate shows why defects occupying a very small fraction of the barrier area can control total radon entry. Consider a 100 m^2^ floor protected by a membrane with a reported coefficient (gas-concentration basis) of *D* = 10^−11^ m^2^ s^−1^ and thickness *d* = 1 mm, with soil-gas concentration *C*_s_ beneath it. Neglecting decay (*d*/*L*_D_ ≈ 0.5, attenuation ≈ 3%), the diffusive entry rate through the intact membrane is *DAC*_s_/*d*, which corresponds to an equivalent volumetric conductance of 1 × 10^−6^ m^3^ s^−1^. Now consider a single unsealed lap of length *l* = 1 m, overlap width *L* = 0.1 m, and effective aperture *w*, subject to an indoor–subslab pressure difference Δ*p* = 5 Pa. Treating the lap as a planar slot, the advective gas flow is

(34) $$Q=\frac{w^{3}l\Delta p}{12\mu_{g}L}$$

and this flow carries radon at approximately *C*_s_. For *w* = 0.1, 0.3, and 0.5 mm, *Q* ≈ 2.3 × 10^−7^, 6.3 × 10^−6^, and 2.9 × 10^−5^ m^3^ s^−1^, so the lap carries about 19%, 86%, and 97% of the total entry while occupying only 1 × 10^−6^ to 5 × 10^−6^ of the floor area. At Δ*p* = 1 Pa, the corresponding shares are still about 4%, 56%, and 85%. Diffusion through the same open lap, *D*_0_*wl*/*L* ≈ 10^−8^ m^3^ s^−1^ for *w* = 0.1 mm, is negligible by comparison; it is pressure-driven flow that makes the defect dominant.

Three implications follow: First, because slot flow scales with *w*^3^, small differences in workmanship produce disproportionate changes in performance. Second, improving the intrinsic coefficient of a membrane beyond the level at which defect flow dominates yields little benefit for the installed system. Third, defect flow is governed by Δ*p*, which explains why barriers are most effective when combined with sub-slab depressurization or other pressure management that reduces or reverses the driving pressure difference. These estimates are consistent with experimental and numerical studies showing that cracks, joints, penetrations, and poorly sealed laps can dominate radon entry into buildings [13,26,27,53].

## 7. Emerging Topics: New Barrier Materials and Thoron-220

Developments in transient inversion, leakage characterization, joint modeling, and scale-model validation have been appraised in Section 3 and Section 4. This section considers two further topics: new barrier materials and thoron-220.

### 7.1. Materials Development

Recent barrier development frequently uses multilayer polymers, metallized films, or filled polymer systems to increase gas resistance while preserving puncture and installation performance. Gas-barrier performance for another molecule does not establish a radon coefficient, however; each product requires a radon-specific measurement with stated partition and boundary assumptions. Publications on generic graphene or nanocomposite gas-separation membranes should not be cited as radon evidence unless ^222^Rn transport was actually measured.

Machine-learning methods could assist in screening material formulations or constructing surrogate models for computationally expensive building simulations. Their use requires sufficiently large and comparable datasets containing consistent definitions of *D_m_*, *D*_eff_, and *D*_app_. At present, differences in apparatus, boundary conditions, and reporting conventions may limit the reliability of data-driven predictions.

### 7.2. Relevance of Thoron-220

Thoron (*Rn*^220^) requires separate treatment rather than simple inclusion in a *Rn*^222^ transport model. Its half-life is approximately 55.6 s [81], compared with 3.82 d for *Rn*^222^ [82,83]. The characteristic diffusion length of a decaying radionuclide is

(35) $$L_{D}=\sqrt{\frac{D}{\lambda }}=\sqrt{\frac{D\;T_{1/2}}{\ln2}},$$

where *D* is the diffusion coefficient, *λ* is the radioactive decay constant, and *T*_1/2_ is the half-life. If the two isotopes are assumed to have the same diffusion coefficient in a given material,

(36) $$\frac{L_{D,220}}{L_{D,222}}=\sqrt{\frac{T_{1/2,220}}{T_{1/2,222}}}=\sqrt{\frac{55.6}{3.82\times 86400}}\approx 1.30\times 10^{-2}$$

Thus, the characteristic diffusion length of *Rn*^220^ is approximately 1.3%, or about 1/77, of that of *Rn*^222^. Thoron transport is consequently concentrated near emitting surfaces and along short pathways [80,81,84,85]. A chamber and tubing arrangement suitable for *Rn*^222^ may lose a substantial fraction of *Rn*^220^ before it reaches the detector. For a source-to-detector transit time *t_tr_*, the surviving fraction is

(37) $$f_{\mathrm{surv}}=\exp\left(-\lambda_{220}t_{\mathrm{tr}}\right)=2^{-t_{\mathrm{tr}}/T_{1/2,220}}\;$$

The measurement system must therefore account for transit decay, tubing volume, flow rate, mixing, and isotope-dependent detector response [85,86,87]. It must also distinguish *Rn*^220^ from *Rn*^222^. The shorter diffusion length suggests that surface treatments may affect thoron exhalation differently from bulk *Rn*^222^ transport, but this effect should be verified for each material and configuration. Studies of radon–thoron exhalation and indoor distributions provide relevant context [80,83,84,85,88]. ISO/TS 11665-13:2025 is specifically a *Rn*^222^ diffusion-coefficient method and should not be presented as a thoron standard [25].

## 8. Implications, Recommendations, and Research Needs

This section combines the research gaps and practical implications identified in the preceding sections. Each subsection states what the evidence implies and the corresponding research need; actionable recommendations for specific stakeholders, with suggested quantitative criteria, are summarized in Table 8. These criteria are proposed by the authors as practical starting points derived from the analyses in Section 2, Section 3, Section 4, Section 5 and Section 6; they are not established regulatory limits.

### 8.1. Measurement Harmonization and Reporting

Comparability of reported coefficients is limited less by the precision of individual measurements than by inconsistent definitions and incomplete reporting (Section 6). Every reported value should identify whether it is *D*_m_, *D*_eff_, or *D*_app_; the governing equation and boundary conditions used to infer it; the test temperature, humidity, and pressure difference; the leakage and background treatment; and an uncertainty budget that includes model-form uncertainty (Section 4.4).

ISO/TS 11665-13:2017 established a two-side activity-concentration test method for determining the Rn^222^ diffusion coefficient of waterproof membranes. It has been superseded by ISO/TS 11665-13:2025 [25]. Authors should identify the edition followed and report any deviations concerning chamber geometry, specimen conditioning, leakage assessment, steady-state criteria, and uncertainty evaluation. Recent membrane and diffusion-cell methodologies include those reported by Ruvira et al. [11], Tejado Ramos et al. [45], Castaño-Casco et al. [46], and Portaro et al. [17]. Because these studies use different apparatus and analysis models, comparison requires clear definitions of the fitted coefficient, boundary conditions, detector response, and leakage treatment.

### 8.2. Joint and Penetration Testing

Priority: high. ISO/TS 11665-13:2025 specifies methods for determining the ^222^Rn diffusion coefficient of waterproofing materials mounted in a test device [25]. However, a material-specimen result does not by itself describe the performance of installed overlaps, seams, penetrations, or repaired regions. Experimental and numerical studies show that joints and localized defects can substantially reduce the overall resistance of a barrier assembly [26,27,35,53].

Research should address:

- Reference geometries for lap joints, butt joints, taped seams, welded seams, corners, and pipe penetrations;
- Procedures for separating transport through the intact material from transport through the joint;
- Acceptance criteria that can be checked during installation;
- Reporting formats for joint geometry, adhesive type, workmanship, pressure difference, and aging condition; and
- Databases of assembly-level measurements for specified membrane–joint–adhesive combinations.

The measured result should be described as an assembly-level apparent coefficient, leakage conductance, or joint transmission rate rather than as the intrinsic diffusion coefficient of the membrane.

Standards bodies should extend material-level testing by developing reproducible procedures for evaluating overlaps, seams, penetrations, repairs, and other construction details [27,35,39]. Such procedures should specify geometry, pressure conditions, environmental conditioning, leakage assessment, detector requirements, calculation methods, and acceptance criteria.

### 8.3. Laboratory-to-Field Translation

Priority: high. A low diffusion coefficient measured using an intact laboratory specimen does not guarantee low indoor radon concentrations after installation. Field performance also depends on joints, penetrations, construction damage, soil-gas pressure, ventilation, room volume, and source distribution [8,26,27,44].

The parallel-pathway estimate in Section 6.5 shows why this is so: a lap or penetration occupying 10^−6^–10^−5^ of the floor area can carry most of the radon entry when it is exposed to a pressure difference of a few pascals. Installed performance therefore depends at least as much on joint integrity and on the driving pressure difference as on the intrinsic coefficient.

Any proposed laboratory-to-field scaling factor requires a defined response variable, applicable conditions, and independent validation.

Radon barriers should normally be considered as one component of an integrated mitigation strategy that may include sub-slab depressurization, sealing of entry routes, and appropriately designed ventilation [4,9,89]. Measurements made under ISO/TS 11665-13:2025 should identify the specific test method and any deviations from the specification [25]. The ISO method applies to waterproofing materials such as membranes, coatings, and paints; it should not be presented as a general method for porous building materials.

Because sub-slab membranes are usually inaccessible after construction, “periodic membrane inspection” is generally impractical. Instead, projects should require documented inspection before covering, followed by post-construction indoor radon measurement. Radon should be measured again after major building alterations, changes to ventilation, foundation work, or failure of a mitigation component.

Finally, radon-control requirements should be coordinated with energy-efficiency policies. Increased airtightness does not inevitably increase radon, but it can reduce dilution or alter pressure-driven soil-gas entry if ventilation and pressure management are not considered. Building policies should therefore assess radon protection, ventilation performance, indoor air quality, and energy demand together [9,89].

### 8.4. Durability

Long-term evidence remains the weakest part of the evidence base. Few studies track how the intrinsic coefficient, seam conductance, and defect-related leakage of installed assemblies change during service, and accelerated-aging protocols have not been linked quantitatively to field failure mechanisms such as thermal cycling, oxidation, plasticizer loss, moisture exposure, loss of adhesion, and mechanical damage [70,71,72,73]. Aging studies should report the intact-material coefficient and the transmission of seams and penetrations separately, before and after exposure (Section 5.3.3), and multi-year monitoring of buildings with documented barriers, installation details, and pressure conditions is needed to test whether accelerated aging predicts service performance.

### 8.5. Interlaboratory Comparison and Standardization

The first international comparison of radon diffusion-coefficient measurements [40] and the subsequent analysis of inter-method differences [41] indicate that harmonized protocols, rather than more sophisticated data analysis alone, are needed to reduce scatter. New round-robin exercises should use common specimens with documented conditioning, common data-analysis files so that apparatus and fitting effects can be separated, blank-holder leakage tests, and harmonized uncertainty budgets consistent with JCGM 100 and JCGM 101 [25,56,57].

Building codes that reference ISO/TS 11665-13 should identify the applicable edition and state whether acceptance is based on *D*_m_, *D*_eff_, *D*_app_, *P*, the area-normalized resistance *d*/*P*, the radon flux transmission, or an assembly-level test. These quantities are not interchangeable, and any numerical limit should specify specimen thickness, temperature, humidity, pressure difference, boundary conditions, and the treatment of measurement uncertainty [25]. Table 8 summarizes the recommended actions by stakeholder.

## 9. Conclusions

The following conclusions are drawn from the reviewed literature:

1. Reported “radon diffusion coefficients” are not equivalent quantities. *D*_m_, *D*_eff_, and *D*_app_ differ in physical meaning; for dense membranes, values obtained from gas-phase concentrations incorporate the partition coefficient and are permeability-type quantities.
2. Protocol differences explain much of the scatter in published values. Identical membranes measured by different methods have differed by up to two orders of magnitude, mainly because of short tests, low source concentrations, and steady-state analysis of non-steady data.
3. The inferred coefficient is sensitive to non-ideal conditions. Inter-chamber leakage adds a concentration-independent term that dominates for the most resistant membranes; receiver loss, gas-side resistance, detector lag, and advection bias the result in identifiable directions.
4. Transport regime and gas-side resistance can be assessed quantitatively. Under typical pressure differences, low-permeability specimens have Peclet numbers far below unity, whereas sub-millimeter cracks and joints can be advection-dominated; gas-side resistance is negligible for dense membranes but not necessarily for porous materials in unstirred chambers.
5. Reported uncertainties are usually incomplete, because model-form uncertainty, which can exceed the statistical component, is rarely quantified.
6. Laboratory coefficients do not by themselves predict installed performance. Pressure-driven flow through laps, joints, and penetrations occupying less than 10^−5^ of the barrier area can dominate radon entry.
7. Thoron requires isotope-specific treatment, because its diffusion length is about 1.3% of that of ^222^Rn. Evidence on long-term durability, joint performance, and interlaboratory comparability remains limited.

## Figures and Tables

**Table 1:** Detector-related issues that affect the inferred diffusion coefficient.

Issue	Why It Matters for the Inferred Coefficient
Response time and cycle time	Can distort transient concentration curves; must be modeled or shown to be short relative to the breakthrough time.
Humidity	Can introduce concentration bias for some detectors (e.g., electrostatic collection); dry or condition the sampled gas, or apply a validated correction.
Calibration	Determines the concentration scale; calibration errors cancel only partly unless source and receiver concentrations are measured with the same calibrated system.
Background	Important for low-flux measurements; a drifting receiver background can mimic specimen transfer.
Sampling, flow, and tubing	Can introduce additional delay, dilution, or losses (and thoron decay); detector and tubing volumes belong in the chamber balance.

**Table 2:** Comparative appraisal of approaches used to determine radon transport coefficients.

Approach	Quantity Obtained	Typical Duration	Main Strengths	Dominant Bias Sources	Best Suited to
Steady-state flux (Equation (18))	*D*_app_, or *P* on a gas-concentration basis	Days to weeks; 2–3 weeks when *d* ≫ *L*_D_	Transparent; no initial profile needed	Steady state not reached; drift; leakage; decay curvature	Porous materials; permeable coatings
Instantaneous exposure (Equation (19))	Lumped rate constant; *D* only via a cell model	Hours to days	Rapid; simple apparatus	Cell-model dependence; partitioning; background offset	Screening and comparative tests
Accumulation (Equation (20))	*k*_eff_ and asymptote; *D* via chamber model	Days	Robust; simple fitting	Multiple time constants; receiver leakage; detector lag	Membranes of moderate resistance
Full transient inversion (Section 4.4)	*D*_app_; *K* and *G*_l_ if identifiable	Days to about two weeks	Uses entire history; can include leakage, detector, and chamber dynamics	Model-form error; parameter correlation; initial conditions	Highly resistant membranes
Joint and assembly tests	Assembly-level *D*_app_, joint transmission, or leakage conductance	Days to weeks	Captures seams and penetrations	Geometry-specific; not transferable to the matrix	Installed-system evaluation
Scale-model room or field study	Indoor concentration reduction or entry rate	Weeks to years	Integrates pressure, ventilation, and workmanship	*D* not identifiable; site specificity	Validation of building-scale predictions

**Table 3:** Coefficient modified by each influencing factor.

Factor	*D*_m_ (Dense Phase)	*D*_eff_ (Porous Medium)	*D*_app_ (Fitted Value)	Section
Porosity, connectivity, tortuosity	Not applicable	Primary	Inherited	Section 5.1.1
Polymer composition, crystallinity, free volume, fillers	Primary (*K* also changes)	Not applicable	Inherited via *P* = *KD*_m_ when gas-phase concentrations are used	Section 5.1.2
Temperature	Primary (Arrhenius; *K* also changes)	Moderate (gas diffusivity, moisture redistribution)	Inherited	Section 5.2.1
Moisture content	Possible (water uptake; formulation-specific)	Primary (gas-filled porosity, partitioning)	Inherited; also via detector humidity response	Section 5.2.2
Pressure difference, advection	None	None (a Darcy term is added)	Biased if advection is unmodeled	Section 5.2.3
Specimen thickness	None	None for a homogeneous specimen	Affected through decay attenuation, identifiability, and defect fraction	Section 5.3.1
Edge sealing, leakage, gas-side boundary layer	None	None	Biased (upward for inter-chamber leakage; downward for receiver loss or gas-side resistance)	Section 3.2.4 and Section 5.3.2
Aging and degradation	Possible (free volume, oxidation, crystallinity)	Possible (microcracking)	Affected, especially through defects and joints	Section 5.3.3

“Inherited” indicates that *D*_app_ reflects any genuine change in *D*_m_ or *D*_eff_; *D*_app_ additionally includes any unmodeled measurement effect.

**Table 4:** Examples of reported radon diffusion coefficients under specified experimental conditions.

Material (Source)	Reported Coefficient (m^2^ s^−1^)	Measurand as Reported	Method and Boundary Conditions	Specimen Thickness	Temperature /RH	Leakage Correction
HDPE, 29 products [38]	1.8 × 10^−12^–2.3 × 10^−11^ (mean 6.1 × 10^−12^)	*D* on gas-concentration basis; includes solubility and adsorption	Czech method K124/02/95: two-chamber; after steady state, receiver flushed and build-up re-measured with source held at 1–100 MBq m^−3^; *D* from iterative numerical solution of the diffusion–decay equation [15,18]; exposed area 1.6 or 2.5 × 10^−3^ m^2^; ≥2 specimens per product	Product thickness; not tabulated	Not reported	Not reported
LDPE, 22 products [38]	2.2 × 10^−12^–5.1 × 10^−11^ (mean 1.8 × 10^−11^)	As above	As above	As above	As above	As above
PVC, 44 products [38]	2.1 × 10^−12^–4.8 × 10^−11^ (mean 1.8 × 10^−11^)	As above	As above	As above	As above	As above
Aluminum-film bitumen, 54 products [38]	1.4 × 10^−15^–5.5 × 10^−12^ (mean 2.9 × 10^−13^)	As above	As above	Product thickness; Al film 0.006–0.08 mm	As above	As above
Polymer–cement coatings, 17 products [38]	1.1 × 10^−11^–5.7 × 10^−10^ (mean 1.3 × 10^−10^)	As above; cured coating (assembly-level)	As above; ≥3 specimens per product	Applied thickness; not tabulated	As above	As above
Polyethylene foil, 1–3 layers [34]	(1.45–2.14) × 10^−12^	*D*_app_	Two-chamber cell after ISO/TS 11665-13:2025 (2 × 0.801 L; 78.5 cm^2^); 1.47 kBq pitchblende source; RAD7 monitors in closed loops; steady-state calculation (ISO Annex A.5) from the last 24 h of a 48 h test; duplicate tests	10, 20, 30 µm (±5 µm)	18.0 °C (variation 1.0 °C); detector inlet dried; specimen RH not reported	Leak rate 0.0034 h^−1^ (≈0.45*λ*), below the ISO limit; assumed negligible, not corrected
Aluminum foil, 1–3 layers [34]	(0.75–1.64) × 10^−13^	*D*_app_; apparatus- or defect-limited (see text)	As above	15, 30, 45 µm (±5–8 µm)	As above	As above
PE–Al–PE and PE–Al–PE–Al–PE [34]	2.1 × 10^−13^ and 3.6 × 10^−14^	Assembly-level *D*_app_	As above	35 and 60 µm	As above	As above
PP, HDPE, LDPE foils [19]	*D*_m_ at 21 °C: 8.2 × 10^−14^, 1.6 × 10^−12^, 3.1 × 10^−12^; *P* = *KD*_m_: 3.0 × 10^−13^, 4.1 × 10^−12^, 1.1 × 10^−11^	*D*_m_ = *λL*_D_^2^ and *K* (2.5–3.7 at 21 °C) determined separately	Absorption in a closed loop (tens of MBq m^−3^, 2–3 d), then desorption into radon-free air; liquid scintillation (Cherenkov) counting; multi-exponential fit; no two-chamber boundary conditions	Foil stacks: PP ≈ 30 µm, LDPE ≈ 75 µm, HDPE ≈ 122 µm	5, 10, 21, 31 °C (±1 °C); RH not reported	Not applicable; exposure-vessel leakage < 1% of activity
Polycarbonate foils (Makrofol N, DE) [19]	*D*_m_ at 21 °C: 2.8 × 10^−15^ and 3.9 × 10^−15^	As above; *K* = 103 and 35 at 21 °C	As above	≈42 and ≈50 µm	As above	As above
HDPE sheet (sample 1) [17]	3.07 × 10^−12^ (configuration 1); 2.54 × 10^−12^ (configuration 2)	*D*_app_ from transient inversion	ISO/TS 11665-13:2017 method A: TESTMAT two-chamber cell (28 cm^2^; receiver circuit 2.84 or 1.39 L); weak non-stationary source (~100 kBq m^−3^); about 2 weeks; measured source and receiver concentrations as boundary functions in an explicit finite-difference fit; Monte Carlo uncertainty (400 scenarios)	1.13 mm	Monitored, values not reported; source circuit dried	Yes: *λ*_F_ = 1.42 × 10^−7^ s^−1^ (≈7% of *λ*) and background (43.6 Bq m^−3^) measured and modeled
HDPE sheet with non-woven fabric (sample 3) [17]	7.38 × 10^−12^	As above	As above (configuration 1)	0.81 mm	As above	As above
Polyolefin (FPO) membrane laminated with PP fabric (sample 2) [17]	1.97 × 10^−11^; 1.19 × 10^−10^ with an unsuitable four-nail holder	As above	As above (configuration 1)	1.30 mm	As above	As above

HDPE, high-density polyethylene; LDPE, low-density polyethylene; PVC, polyvinyl chloride; PE, polyethylene; PP, polypropylene; FPO, flexible polyolefin; RH, relative humidity. Values from [38] are category ranges with means in parentheses; ranges describe product variability, not measurement uncertainty, and the coefficients incorporate solubility and adsorption [38]. For HDPE, the gas-basis coefficients from [38] and [17] should be compared with *P* = *KD*_m_ from [19], not with *D*_m_. For the FPO membrane of [17], other laboratories in an intercomparison reported 3.5 × 10^−12^–1.2 × 10^−11^ m^2^ s^−1^. Values are not directly comparable across rows (see text).

**Table 5:** Minimum data needed to compare dense-membrane results.

Category	Required Information	Interpretation Risk If Omitted
Specimen	Polymer grade, reinforcement, thickness at several points, conditioning, exposed area	Product name alone does not define the tested material
Transport quantity	*D_m_*, *D_eff_*, *D_app_*, *L_D_*, *P*, diffusion length, or RFT	Different quantities may have the same reported unit or symbol
Interfaces	Gas- or material-phase concentration; partition condition; air-side transfer	A fitted value may absorb solubility or boundary resistance
Apparatus	Chamber volumes, pressure difference, seal and blank leakage, detector response	Fixture transfer can dominate a highly resistant specimen
Result	Estimate, standard/expanded uncertainty, detection limit, replicate variation	Values near the leakage-equivalent limit may be only upper bounds

**Table 6:** Minimum data needed to compare porous-material results.

Category	Required Information	Interpretation Risk If Omitted
Microstructure	Density, total and connected porosity, mix or product composition, curing/age	Nominal material class conceals large batch differences
Moisture	Gravimetric or volumetric water content, RH conditioning, saturation definition	“Dry” and “wet” are not reproducible states
Scale	Specimen dimensions, representative-volume justification, cracks and edge sealing	Small cores may exclude dominant field pathways
Model	Gas-phase or multiphase governing equation, definition of *D_eff_*, and treatment of radon production, emanation, and decay	The fitted coefficient can combine diffusion and production
Regime	Pressure difference, permeability or velocity, Peclet number	Advection may be mislabeled as diffusion

**Table 7:** Minimum data needed to compare coatings and assemblies.

Category	Required Information	Interpretation Risk If Omitted
Application	Wet and cured thickness, number of coats, cure conditions, substrate preparation	Nominal coverage does not establish diffusion path length
Defects	Pinholes, cracks, overlaps, penetrations, edge seal, inspection method	Bypass flux can dominate the continuous coating
Aging	Thermal, moisture, mechanical and chemical exposure before/after testing	Initial coupon performance may not represent service life
Metric	Material *D_m_*, assembly *D_app_*, P (permeability), RFT, or exhalation reduction	A surface-flux reduction is not necessarily a diffusion coefficient
Validation	Replicates, blank holder, alternate thicknesses, field or scale-model comparison	Model-form bias can remain hidden in a single configuration

**Table 8:** Actionable recommendations by stakeholders, with suggested quantitative criteria.

Stakeholder	Recommended Action	Suggested Criterion or Check
Researchers and testing laboratories	Define the measurand (*D*_m_, *D*_eff_, or *D*_app_) and report model, boundary conditions, temperature, humidity, and pressure difference	Complete reporting set of Table 5, Table 6 and Table 7; at least three replicate specimens
	Verify fixture leakage with blank-holder or sealed-fixture tests	*D*_leak_ = *G*_l_*d*/*A* below 10% of the expected *D* (Section 3.2.4); otherwise fit *G*_l_ or report an upper bound
	Confirm the transport regime	Measured or bounded Δ*p*; *Pe* = *k*_g_Δ*p*/(*μ*_g_*D*_bulk_) ≤ 0.1 for a diffusion-only analysis (Section 2.4)
	Verify gas-side boundary conditions	*Bi*_m_ ≥ 200 to neglect gas-side resistance; Robin conditions if *Bi*_m_ ≤ 20 (Section 5.3.2)
	Demonstrate steady state or use transient inversion	Pre-defined flux criterion; for *d* ≫ *L*_D_, allow two to three weeks (Section 3.3)
Modelers	Verify numerical models and quantify model-form uncertainty	Grid and time-step convergence; stated mass-balance residual; multi-model spread included in *u*_c_ (Section 4.4)
Manufacturers	Declare the transport metric with its test conditions and uncertainty; supply joint and penetration data	Coefficient with thickness, temperature, RH, method edition, and expanded uncertainty with stated *k*; assembly-level data for recommended joints
Designers and installers	Specify overlaps, joint sealing, penetrations, horizontal–vertical connections, puncture protection, and repairs; inspect before covering	Documented pre-cover inspection; post-construction indoor radon measurement against the national reference level (100–300 Bq m^−3^ [4,5])
Standards bodies and regulators	Develop joint and penetration test methods; organize interlaboratory comparisons; state the regulated metric	Reference joint geometries; round-robin with common specimens and data files; limits specifying metric, thickness, temperature, RH, and Δ*p*

## Data Availability

No new data were created or analyzed in this study. Data sharing is not applicable to this article.

## Abbreviations

The following abbreviations are used in this manuscript:

ISO	International Organization for Standardization	
WHO	World Health Organization	
RFT	Radon flux transmission factor	
TESTMAT	The name of the experimental setup	
ENDORSE	The dedicated software name	
PVC	Polyvinyl Chloride	
FEM	Finite Element Method	
MC	Monte Carlo	
GUM	Guide to the Expression of Uncertainty in Measurement	
JCGM	Joint Committee for Guides in Metrology	
CFD	Computational fluid dynamics	
Bi	Biot number	
Pe	Peclet number	
Nomenclature		
Symbol	Description	Unit
*A*	Exposed specimen area	m^2^
*C*, *C_g_*, *C_m_*	Radon activity concentration; gas-phase and material-phase concentrations where distinguished	Bq m^−3^
*C_sc_*, *C_rc_*	Bulk activity concentration in source and receiver chambers	Bq m^−3^
*D_m_*	Molecular or material diffusion coefficient in a homogeneous phase	m^2^ s^−1^
*D_eff_*	Effective diffusion coefficient of a porous or heterogeneous medium	m^2^ s^−1^
*D_app_*	Apparent coefficient inferred from a specified experiment and model	m^2^ s^−1^
*D*_0_	Diffusion coefficient of radon in free air (approximately 1.1 × 10^−5^ at room temperature)	m^2^ s^−1^
*d*	Specimen thickness	m
*h*	Air-side mass-transfer coefficient	m s^−1^
*J*	Radon activity flux	Bq m^−2^ s^−1^
*K*	Material/gas equilibrium partition coefficient, $\frac{C_{m}}{C_{g}}$	1
*k_l_*	First-order chamber leakage or air-exchange rate	s^−1^
*L_d_*	Diffusion length, $\sqrt{\frac{D}{\lambda }}$	m
*P*	Permeability coefficient, *KD_m_* for a dense membrane	m^2^ s^−1^
*Pe*	Peclet number, $\frac{\left|u_{D}\right|L_{c}}{D_{\mathrm{bulk}}}$	1
*S*	Volumetric radon source term	Bq m^−3^ s^−1^
*t*	Time	s
*u_c_*	Combined standard uncertainty	same unit as measurand
*V_s_*, *V_r_*	Source- and receiver-chamber volumes	m^3^
*x*, x	Spatial coordinate	m
*ε*	Connected gas-filled porosity	1
*λ*	Radionuclide decay constant	s^−1^
*τ*	Tortuosity factor under the stated convention	1
*D_bulk_*	Bulk-area gas transport coefficient; equal to *D_eff_* under the porous convention used here	m^2^ s^−1^
*D_pore_*	*D_bulk_*/*ε_g_* for constant gas-filled porosity	m^2^ s^−1^
*u_D_*; *v_g_*	Darcy velocity; pore-gas velocity, *v_g_* = *u_D_*/*ε_g_*	m s^−1^
*D_pre_*	Arrhenius pre-exponential factor	m^2^ s^−1^
*Bi*_m_	Mass-transfer Biot number, *hd*/*P*	1
*D*_leak_	Leakage-equivalent diffusion coefficient, *G*_l_*d*/*A*	m^2^ s^−1^
*G*_l_	Calibrated inter-chamber leakage conductance	m^3^ s^−1^
*k*_g_	Gas permeability	m^2^
